# The Brain Is Adaptive Not Triune: How the Brain Responds to Threat, Challenge, and Change

**DOI:** 10.3389/fpsyt.2022.802606

**Published:** 2022-04-01

**Authors:** Patrick R. Steffen, Dawson Hedges, Rebekka Matheson

**Affiliations:** ^1^Department of Psychology, Brigham Young University, Provo, UT, United States; ^2^Departments of Psychology and Neuroscience, Brigham Young University, Provo, UT, United States

**Keywords:** adaptive, triune, prediction, stress response, brain

## Abstract

Theory impacts how research is conducted. A popular theory used to conceptualize brain functioning is the triune brain theory. The triune brain theory is an evolutionary theory of brain development that emphasizes three key brain regions consisting of the brainstem, the limbic system, and the cortex that function relatively independently in coping with stress via fight or flight, emotion, and cognition, respectively. However, modern neuroscience research demonstrates that the triune brain theory does not accurately explain how the brain functions in everyday life or during the stress response. Specifically, emotion and cognition are interdependent and work together, the limbic system is not a purely emotional center nor are there purely emotional circuits in the brain, and the cortex is not a purely cognitive center nor are there purely cognitive circuits in the brain. We propose a new evolutionarily based model, the adaptive brain, that is founded on adaptive prediction resulting from interdependent brain networks using interoception and exteroception to balance current needs, and the interconnections among homeostasis, allostasis, emotion, cognition, and strong social bonds in accomplishing adaptive goals.

*“We do not live to think, but, on the contrary, we think in order that we may succeed in thriving.”*

—Jose Ortega y Gasset

## The Triune Brain: an Outdated, Inaccurate Model

Theory impacts how research is conducted. An influential theory used to conceptualize brain function and drive research has been the triune brain theory (1–4). The triune-brain approach to understanding the brain takes an evolutionary perspective about how the brain has developed under environmental pressures and how that development impacts our responses, particularly our responses to stress. Describing the triune-brain theory, MacLean (3) states that:

The human forebrain evolved to its great size while retaining features of three basic formations that reflect an ancestral relationship to reptiles, early mammals, and recent mammals. The three neural assemblies… are radically different in structure and chemistry, and in an evolutionary sense, countless generations apart. Psychological and behavioral functions depend on the interplay of three quite different mentalities. The three evolutionary formations might be popularly regarded as three interconnected biological computers, each having its own special intelligence, its own subjectivity, its own sense of time and space, and its own memory, motor, and other functions.” (p. 264).

From the perspective of the triune-brain theory, these three brain regions evolved separately and function somewhat independently: the basal ganglia and brain stem are involved in movement and basic life functions, the limbic system is involved in emotional responses that are seen more prominently in mammals as compared to reptiles, and the cortex is involved in cognition and executive functions and is most prominent in humans. In this perspective, evolutionary development begins with basic behavioral responses, then adds emotional responses that can alter these basic responses when threat or challenges arise, and then adds on cognition to alter emotional responses using reason, logic, and planning.

There are several key problems, however, with the triune brain theory. First, the brain did not evolve in successive stages as MacLean hypothesized (5). The idea that vertebrate evolution has consisted of “newer brain structures being superimposed over and on top of ‘older' brain structures, tracking development of complex cognition,” is not evolutionarily justifiable (6). Basic neural regions are shared among all vertebrates. Where they differ is in proportion and extent. Just as an elephant's trunk is not a new structure superimposed over a snout but rather is an analogous structure differing in proportion (and consequently, in functional adaptation to the animal's needs), the human brain is not superimposed on a reptile brain but consists mostly of proportionally different analogous structures. Furthermore, the gradation of proportional shifts is not necessarily a linear progression from reptile to human (7).

Second, brain structures do not function independently of one another (5). During emotional responses, there is activity in the amygdala and in the limbic system, but there is also activity in cortical areas and in the brainstem (8). Additionally, emotion and cognition are not independent events; rather, they are interrelated functions working in concert. For example, Bush et al. (9) and Shackman et al. (10) both note that emotional responses and cognitive responses in the cingulate cortex are interconnected and not separate as previously believed. Perhaps more importantly, the limbic system is not a purely emotional center in the brain. LeDoux (8) notes that the hippocampus is considered part of the limbic system but that it is not considered an essentially emotional brain region; instead, it is a key area involved in memory, which is more closely associated with cognition. Because of these and similar problems, the term “limbic system” is no longer a commonly used term to describe how the brain functions. “Limbic system” also loses its utility in a clinical setting; because affect is a culmination of a wide range of interrelated processes, including synthesis of internal and external stimuli, arousal, and memory, approach to disorders characterized by affect dysregulation is limited by the triune brain approach. And, finally, the brain does not act by simply responding to a stimulus. Instead, it predicts internal and external needs and adapts accordingly. Incoming stimuli interact with the current state in which the brain is (11).

Third, current neuroscience research findings provide further evidence of the inaccuracy of triune brain theory and open new ways of understanding how the brain responds to stress and adapts to changing internal and external environments. Fear research provides an instructive specific example. There is no fear brain circuit that turns on during a fear response but otherwise lies dormant (8). Brain networks always have some level of activity (12) that affects how they process incoming information (11). What changes is the relative activity of different brain networks, with networks being differentially activated based upon need (13–16). As these findings show, triune-brain theory does not match current research findings and using triune-brain theory as a general theoretical approach can lead to faulty hypothesis creation and poorly developed studies.

A more useful evolutionary theory of how the brain works needs to integrate accurate knowledge of brain structure and function. Adaptation, survival, and reproduction are at the heart of evolutionary theory, and interdependent brain networks have evolved to increase adaptation to be able to survive and reproduce. Further, emerging findings suggest that the brain uses interoceptive and exteroceptive information to predict future conditions and needs to enable optimal adaptation to continuously changing internal and external environments (11–20). Based on better understanding of how the brain works, we propose replacing “triune brain” with a term that better captures current understanding of brain function: the adaptive brain. In this conceptualization, the term adaptive brain emphasizes the interdependence and plasticity of brain regions and the brain's ability to predict and adapt to future needs and conditions. Instead of three relatively independent brain regions, or any number of independent brain regions, brain networks work together interdependently; instead of purely “emotional circuits” or “cognitive circuits,” the brain uses interconnected networks to optimize maintenance of the body's internal state, emotion, and cognition to adapt to continuously changing needs (11). The brain's summated approach to these priorities regulates affect, and dysregulation of these interdependent circuits has important implications for psychopathology.

## The Adaptive Brain: Prediction, Balance, and Interdependent Brain Networks

The adaptive brain developed out of millions of years of evolutionary pressure. Throughout most of their evolutionary history, humans have existed in hunter-gatherer bands, and the evolutionary pressures experienced occurred in this context. This developmental period has been termed the “Environment of Evolutionary Adaptation” because our current adaptations are a direct result of this experience (21). Some of the most important evolutionary pressures experienced were limited resources and dangerous environmental conditions such as predators and extreme weather, and the brain evolved to predict the most adaptive course of action accounting for limited resources and danger, balancing internal needs and external demands (22, 23).

Allostasis, or stability through change that depends on predicting future needs and conditions (17), emphasizes our ability to anticipate and adapt to diverse environmental forces to balance internal needs and external demands (22, 24). Schulkin and Sterling (23) argue that allostasis is basically brain-centered predictive regulation. The brain is continuously evaluating our internal and external environments based on previous experience, predicting what is likely to occur, and then determining the best course of action based upon this available data. Allostasis, therefore, is about adapting to changing internal and external environments with the goal of stability even when faced with uncertain circumstances, balancing internal parameters essential for life with the changing world around us. Sterling (25) notes that the goal of allostatic balance is not constancy but fitness under the conditions of natural selection. The goal of all organisms is not constancy but survival to reproduce, and the goal of adaptive regulation is reproductive success. Adaptive balance and regulation result from developmental trajectories designed to optimize successful competition.

Challenges, opportunities, and threats can appear quickly requiring rapid responses. Perhaps the most important adaption that evolved during the Environment of Evolutionary Adaptedness is the brain's ability to simulate and predict potential outcomes in coping with challenge and threat (26–28). Predicting likely outcomes increases speed and efficiency of response and improves the brain's adaptivity. To increase the power of prediction and subsequent adaptivity, the brain works to minimize prediction errors; that is, minimizing the difference between predicted outcomes and actual incoming interoceptive and exteroceptive information. The more the brain can minimize prediction error and accurately predict outcomes for different courses of action, the better it will be at anticipating and adequately responding to challenge and threat efficiently and rapidly, thus increasing adaptation and survival (20, 29, 30). Cutting across previously accepted boundaries of the triune brain, Barrett and Simmons (29) propose a neuroarchitecturally distinct brain interoceptive system consisting of visceromotor cortex in the medial and anterior cingulate cortex, the posterior ventromedial prefrontal cortex, the posterior orbitofrontal cortex, and the anterior insular cortex (31). This interoceptive system transmits information through connections in the amygdala, hypothalamus, ventral striatum, and periaqueductal gray to the spinal cord that predicts needed autonomic, hormonal, and immunologic adjustments. According to Barrett's and Simmons' proposal, the frontal interoceptive center also sends this same information to the mid and posterior insula, which can then determine the prediction error to maintain optimal energy use, or homeostasis (29) or initiate allostasis by ongoing adaptation to changing internal and external environments, including internal energy states (17).

Active inference approaches further emphasize the importance of brain adaptivity. These approaches to understanding brain adaptation incorporate prediction and the importance of minimizing prediction errors but also include investigation of how the brain predicts outcomes of different possible behaviors (32, 33) to minimize prediction errors (34). Paulus et al. argue that “the goal of [active inference] is to generate the most complete model of the world to help guide the most adaptive behavior…” [(35), p. 100].

The importance of prediction and minimization of prediction error in brain function has important implications for brain organization. If the brain is not organized in distinct and functionally independent regions, then how is it organized? The organization of the brain reflects the fact that adaptation and survival depend on effectively balancing and predicting often-conflicting needs. Internal needs (i.e., food) must be balanced with external demands (i.e., not being eaten, fight or flight, as well as everyday stressors). The adaptive brain must be able to respond to stress quickly *and* rationally; depending on the context, speed, including automation of response, may be a greater priority than a careful consideration of several outcomes, or vice versa. Our very survival can depend on our ability to change our current course of action to respond to potentially advantageous or threatening events (14), and virtually all situations require an integration of internal and external needs, speed, and rationality. Indeed, internal needs vs. external demands and automated rapidity vs. slower deliberation form key axes informing behavior, and these axes are reflected anatomically.

Fox et al. (15) argue that the brain is organized in interdependent networks along *interoceptive* and *exteroceptive* axes. To best predict need, the brain integrates interoceptive information, or awareness of internal functioning such as blood pressure and heart rate, with exteroceptive information, or awareness of the external environment. As the predicted needs of the moment demand, the brain can then quickly reorient its attention between internally and externally directed activities. For example, the interoceptive system informs response to hunger, temperature, illness, or serum sodium concentration. Through the exteroceptive system, we know if there is food available (an opportunity) or if a predator is looking at us as food (a threat). If we become aware that food is available through our exteroceptive systems, we can put our energy into obtaining that food to meet an existing or predicted internal need. If a predator threatens to eat us, however, we will deprioritize our need to eat and instead focus our energy into fight or flight. Our ability to respond to, and coordinate, attention to external vs. internal stimuli is crucial to survival.

Even when the brain is not attending to an external stimulus or an externally defined task, the brain's networks are active. Historically, however, neuroimaging research has treated a brain that is not attending to external stimuli or an externally defined task as an inactive brain—a baseline to compare activity against. This view of an inactive, unengaged brain (when it is not directly attending to external stimuli), however, is inaccurate, and ignores the interoceptive axis of network organization. Because information from the interoceptive systems informs our brains of internal states and needs such as serum glucose concentration, heart rate, and inflammatory state (29), internally directed “tasks” are ever-present, and the brain is ever active, predicting needs and allocating resources differentially for externally vs. internally motivated tasks. Further, activity typical of “rest,” or—more accurately, typical of internally-directed behavior—features distinct recognized patterns on functional neuroimaging. An example of broad network, coordinating activity across historically distinguished “triune” areas, is the default mode network (DMN). Because “rest” is a sophisticated state of coordinated network activity, rather than the absence of activity, it too has the agility to adapt readily to shifting internal needs, or to rapidly adapt to the sudden presence of external needs. Were “rest” to simply be an “off” state, there would be little room for adaptation within it. In addition, were interoceptive functions to be handled by relatively isolated, much less relatively primitive, modules, it is difficult to explain the complexity of shifting behaviors addressing interoceptive change and how those behaviors can overlap with, and modify, behaviors addressing exteroceptive conditions.

Periods of coordinated activation of the DMN are instructive about the brain's shifting focus between internally and externally directed behavior. The DMN is often framed as a “task negative” network, in that it is primarily active in the absence of an externally defined task, including during periods of wakeful rest and/or attention toward self-oriented and social cognition. Its activity is associated with the subjective state of mind wandering and is suppressed for goal-directed, externally oriented cognition characterized by activation of “task-positive” networks such as dorsal and ventral attention networks (13). “Task-negative” and “task-positive” terminology, however, fails to fully reflect the coordination of these sets of networks along interoceptive and exteroceptive *axes* rather than linearity. The brain is not a binary toggle switch, interacting with its environments using its attention networks—or not. In reality, DMN functions are never turned off; instead, these functions are carefully enhanced or attenuated depending on need (12). Rather than use a framework that considers only externally directed cognition as “task,” the interplay between DMN and attention networks reflects shifts between internal and externally motivated tasks, both of which are important for survival and both of which are subject to evolutionary pressures (36). Changes in DMN and attention-network cooperativity or reciprocity are associated with maladaptation, including affect dysregulation and affective disorders (37, 38).

Major functional nodes of the DMN include the posterior cingulate cortex and precuneus; the medial prefrontal cortex; and the angular gyrus, with dorsal medial and medial temporal subsystems. These subsystems are themselves broad, expanding from dorsal medial prefrontal cortex to the temporoparietal junction (for the dorsal medial subsystem) and from the hippocampus to the posterior inferior parietal lobe (for the medial temporal subsystem).

Interestingly, though patterns of activation across these areas are typical of “task-negative” activity, there is remarkable overlap across nodes with “task-positive” activity. Here, the example of the midcingulo-insular (M-CIN) and Central Executive (CN) networks is particularly instructive. The M-CIN is often known by an alternate, functionally descriptive name—the salience network (SN)—and, indeed, it is involved in perception of and attentional regulation toward salient stimuli. It is essential to social behavior, including communication; but it is also essential to self-awareness, including integrating interoceptive function. Both the DMN and the M-CIN make extensive use of parietal and temperoparietal structures, and the M-CIN may act as a regulatory switchboard between prioritized use of the DMN or the Central Executive Network (CEN), used for high-cognitive load tasks (39).

A network model of brain activity makes clear that brain “areas” neither behave in isolation nor take charge of tasks which are easily circumscribed into distinct roles. If each area of the brain is active according to the priority of its singular role, the brain is limited in sophistication to the number of possible combinations of active areas. Instead, if each area instead has a very broad range of possible contributions, all modified and molded by the areas with which it is constantly interacting, their functional potential is dramatically expanded. Moreover, they are adaptable, recruited in a wide variety of changing circumstances and in turn recruiting other areas, always participating in the networked brain in novel, and changing ways.

The case of medial temporal lobe structures, specifically, illustrates the limitations of a triune model. Under the triune brain model, these structures are considered paleomammalian and to a large extent functionally separable from neomammalian neocortex. In fact, medial temporal-lobe structures are fully integrated into both task-negative and task-positive systems and across functions historically ascribed to “reptilian,” “paleomammalian,” or “neomammalian” capability. Indeed, the medial temporal lobe structures share with neocortex their scaffolding of glutmatergic neurotransmission, with circuits evolved to be capable of immediate and dramatic long-term potentiation and long-term depression. Involvement of medial temporal-lobe structures communicating with neocortex is a hallmark of the adaptability of networks—at both molecular and cognitive levels.

Internally focused and externally focused networks also enable the brain to operate in different quadrants of speed and reason. Highly predictable situations require less analysis of external factors to efficiently choose a low-risk behavioral strategy. The DMN enables fast, automated responses to routine situations with learned rules (40). Attention and control networks, in contrast, prioritize increased analysis of external cues and enable slow response systems for situations with harder-to-predict outcomes and fewer or no established rules. The adaptive brain's ability to differentially prioritize these strategies is an instructive example of its overall strategy to assess and address changing needs.

Indeed, whether the adaptive brain's allocation of resources favors activity of the DMN, attention networks, or a combination in any given situation reflects its function as a *predictor* of both the internal and external environment, enabling selection of strategies to maintain homeostasis or to initiate allostasis when needed. Accumulating evidence suggests that the brain uses Bayesian statistical principles to predict environmental states and outcomes based on previous information that the brain has received (20, 30). Structures involved in the brain's Bayesian-like prediction are also implicated in integrating, or differentially prioritizing, brain networks and their adaptive strategies. The insular cortex, for example, has several fundamental roles that seem disparate under a triune-brain model but in fact shed important light on the nature of the adaptive brain. Insular cortex is primary interoceptive cortex activating in response to interoceptive and other stimuli such as self-awareness, pain, heartbeat, gastrointestinal distension, is a key predictive center, and far from functioning in isolation acts as a switch plate or integration center for brain networks (41). Unification of interoceptive, predictive, and integrative roles in centralized control hubs such as the insula enables brain adaptation to changing environmental and internal circumstances and needs to maintain homeostasis and initiate allostasis. In that it might be part of both the executive-control and emotional-salience networks, the insula might be involved with integrating cognition and emotion (41) in its role in adaptivity. In essence, the brain's interoceptive center promotes adaptation to ever changing internal and external environments through prediction and subsequent adjustment.

Information about the past internal and external environments is used to make predictions about what these environments will be like to adapt to changing internal and external environments (29). As Van den Bergh et al. (20) write, “Prediction signals from models in the brain are matched with sensory input, resulting in prediction errors that are fed back to improve the adaptivity of these models when making perceptual inferences and actively navigating the environment [(20), p. 228].” If the brain's predictions are not correct, it orchestrates adjustments to minimize the difference between what it predicts and the ongoing interoceptive and exteroceptive information it receives. This process of making predictions and initiating adjustments to minimize the differences between prediction and the actual information it receives through the interoceptive system involves both granular and agranular cortices (29). The ongoing process of predicting internal states, receiving updated information about internal states, and adjusting to minimize the differences between prediction and current information enables the brain to anticipate and adapt to regulate changing internal environments, such as heart rate, blood pressure, serum electrolyte concentrations, and levels of glucose and carbon dioxide. As Van den Bergh et al. (20) further note, “according to the predictive-processing framework, a basic task of the brain is to construct an adaptive model of the (external and internal) world, although its only source of information to do so is the spatial and temporal patterning of its own internal activity (p. 229).”

As the role of prediction and prediction errors in homeostasis and allostasis suggests, the adaptivity of the brain to changing circumstances can be rapid. For example, in studies of non-human primates, some neurons in the orbitofrontal cortex make predictions about rewards associated with stimuli. A visual stimulus might predict a certain taste, to which a neuron has assigned a value. When the stimulus no longer predicts the reward, that is, when the prediction is in error, neurons rapidly adapt and no longer propagate the error. Remarkably, this adaptation to the altered association between stimulus and reward can occur in as few as 5 s (42), providing the brain with a fast and continually updating prediction strategy that enables rapid adaptation to changing circumstances. Rolls (42) notes that this rapid change in learning associations between a stimulus and its value has important implications for changing behavior when “expected reinforcers are not obtained, in, for example, feeding, emotional, and social situations (p. 62),” an observation emphasizing the importance of prediction and correction of prediction mistakes in adapting to a continually changing environment (26).

In conclusion, successful human evolution results from successful responses to threat and challenge. A core function of the adaptive brain is to manage the stress response when coping with threat and challenge. We increase survival success by adapting to environmental conditions, which include limited and inconsistent resources, competition for those limited resources, and predators. Successful adaptation involves balancing our time and energy between internal needs (e.g., eating to get more energy) and external demands (e.g., flight/flight to cope with predators and competitors). Therefore, successfully competing for limited resources involves acting in a fast and frugal manner. We need to respond quickly in case of an unexpected attack or opportunity. And we need to be frugal with our energy because consistent meals are not guaranteed limiting caloric availability. The brain's focus on minimizing prediction error and enhancing successful responding has developed to help us be fast and frugal.

## Emotion, Cognition, and Social Bonds: Three Solutions to Increasing Adaptation

Three key adaptations that have developed over human evolution to improve prediction and response are quick emotional responses, slower cognitive responses, and seeking others' help to cooperatively respond to the stressor (21, 43–46). As the brain predicts the best available course of adaptive action, it engages these response systems to enable quick, intelligent, and cooperative responses to life threats and challenges. Brain networks work together interdependently to carry out these adaptations, and all three of these responses work together in an integrated, interdependent manner to increase adaptation (47).

Using the strategic vs. tactical response model of Lang et al. (48), where broad strategies refer to approach and avoidance strategies in general and local tactical responses refer to specific actions taken such as freezing versus fleeing when under threat, the three adaptive response systems of emotion, cognition, and social connection represent broad response strategies, and specific tactical responses in any given situation can be many and varied. Lang et al. (48) note that our strategic state “differentially primes or inhibits subsequent behavior” and the interaction of internal and external information over time provide the “background framework for transactions between the organism and its environment (p. 380).” We are always in some state of affect, cognition, and social connectedness, and our current state impacts how we respond to arising threats and challenges (47, 49). If our current affective state is negative, we are more likely to respond in a defensive manner. Our current negative affective state can adversely impact our cognition and increase the likelihood of a defensive response. And if our perception of our current social connectedness is negative, we are more likely to respond in a defensive manner.

### Responding Quickly to Stress

Affect is a representation of how we value our current situation, and our affective reactions arise from whatever we are currently focusing on (50). There are many types of situations that all animals encounter and confront, many different types of threats, challenges, and opportunities that can lead to gain or loss. Clore and Huntsinger (50) argue that affect and emotions are the embodied representations of how we evaluate and value our situation in ways that are adaptive for the species. Russell (49) argues that affect is how we feel at any given point in time, a combination of valence (pleasant to unpleasant) and arousal (low energy to high energy) (49). Lang et al. (48) argue that affect is more than just a current feeling state; rather, it is a broad strategic approach to coping with life in terms of valence and arousal.

Affect is impacted by the activation of neural circuits that evolved to ensure survival (44, 49). These “motive circuits” or “survival circuits” evolved to address the key needs of avoiding what is dangerous and approaching what is beneficial. Our motivation arises from these circuits, and motivational arousal is the foundation of emotion. Dangerous situations elicit unpleasant affect and beneficial situations elicit pleasant affect, and people usually choose behaviors that increase pleasurable outcomes and decrease unpleasant outcomes. Therefore, our decisions involve predictions of future affect (51). That is, our choices are guided by the expected impact they will have on our affective state. In a sense, “positive and negative affect serve as ‘go' and ‘stop' signals” [(52), p. 80] for our current decision making.

Our affective arousal results from the intensity of the motivational need that is determined by the degree of danger or benefit (44). Whereas, emotions come and go, we are always in some state of affect. Our core affect results from the integrated awareness of our internal and external worlds, the integration of interoceptive and exteroceptive information (43). Our current affect is like a “‘neurophysiological barometer' of our relationship of our internal and external environments at a given point in time.” [(43), p. 5].

Emotions, according to LeDoux (8) and Barrett (11), are not what most people think they are. Rather than having dedicated emotion circuits, such as a “fear circuit,” emotions are constructed from what LeDoux (8) calls “survival circuits” [see also (44)]. Survival circuits are wired to address basic life needs such as nutrient and fluid regulation, thermoregulation, and defense against harm. LeDoux and Damasio (53) argue that emotions are integrated physiological responses occurring to meet a significant challenge, whereas feelings are the conscious awareness of these physiological responses. From an adaptation perspective, emotions are fast response patterns that allow us to meet a threat or challenge in minimal time, representing an integrated brain response to meet a specific need. There is no “fear circuit” lying dormant in the brain until a threat appears. Rather, interdependent brain networks respond in an integrated manner to meet a basic need, and we experience this as feeling (11).

### Responding Intelligently to Stress

When coping with current stressors, it is adaptive to remember past challenging or threatening events that might be like the current situation. Cognition is about gaining, representing, and using knowledge. Hagen and Symons (54) argue that the cognitive mechanisms we have today are evolved adaptations that allow us to solve life challenges. In terms of successful adaptation, cognition is about remembering past events and experiences and then using that knowledge to effectively cope with current environmental challenges. In this sense, cognition is basically about problem solving using existing knowledge to adapt more successfully. With cognition, we imagine possible future events and then plan for possible courses of action to cope more effectively with those possibilities.

Cognition works with emotion in meeting needs. Cognition integrates with emotional responses by including knowledge and experience from previous encounters with similar situations. In terms of brain networks, Raichle (12) compares emotion and cognition to Kahneman's ideas of thinking fast and thinking slow, having quick immediate responses and slower thought-out responses. Cognition and emotion are not independent or conflicting responses; instead, they work together toward the same goals (47). Affect impacts decisions through personal values impacting current mental content (50). Positive and negative affect contribute positive or negative value to whatever might be in the mind at the time. Being happy or sad influences the content and focus of thought, with positive affect validating and negative affect invalidating cognitions (50). Our judgments reflect our current affect, with our core affect resulting from the integration of interoceptive and exteroceptive information (43). Therefore, all our mental states are inseparably interconnected with affective content.

Barrett and Bliss-Moreau (43) note that much of the core affective circuitry of the brain was until recently considered cognitive circuitry. Brain networks integrate exteroceptive and interoceptive information to create an integrated representation of our world now. This integration is like a large-scale neural reference space that presents a neural map of our external and internal worlds built on available sensory information (43). This map is then used to predict best courses of action. Barrett and Bliss-Moreau (43) argue that this core affect neural reference space contains two functional networks: one a sensory integration network that is dependent on values and experience and how current environments might impact homeostasis, and the other a visceromotor network that guides responses via autonomic and endocrine functioning.

### Responding Cooperatively to Stress

Finally, strength in social bonds and being able to work with others increases adaptation. Strong social bonds are a key adaptation that developed during the Environment of Evolutionary Adaptation (21). An important problem that early humans likely faced in surviving and reproducing was establishing cooperative relationships (55). Successful human groups were those that most effectively established these cooperative relationships. Being a member of a group can serve many adaptive functions. Evolutionarily, achieving acceptance and status led to better protection, food, and mates, and helping others increases inclusive fitness, with research showing a strong gradient of helping others based on degree of genetic relatedness (55). Interestingly, modern personality theory emphasizes that social acceptance and social status are key foundational principles in personality (56). We all have a desire to “get along and get ahead” (57). We have evolved psychological mechanisms to avoid being excluded, and the need to belong continues to be a central human motive today (58).

Emotions, cognitions, and strong social bonds have evolved together to maximize the stress response and adaptation. LeDoux and Damasio (53) state that “unconscious emotional states are automatic signals of danger and advantage, whereas conscious feelings, by recruiting cognitive abilities, give us greater adaptability in responding to dangerous and advantageous situations. Indeed, both emotions and feelings also play a major role in social behavior, including the formation of moral judgments and the framing of economic decisions” (p. 1,092). Emotions, cognitions, and strong social bonds are not in competition with each other; in contrast, these adaptations work together to maximize how we cope with stress. Without these adaptations, it is unlikely we would be where we are today.

## The Adaptive Brain: Applications and Implications

The brain's organization based on functionally interdependent networks, integration of interoception and exteroception, social bonds, and prediction and minimization of prediction errors indicate that a primary function of the brain is adaptation to internal and external environments in a continual process to maintain homeostasis and implement allostasis as needed. Conceptualizing the brain as an entity one of whose main functions is adaptation has both theoretical and clinical implications.

### Theoretical Implications

Viewing the brain as an extraordinarily integrated and adaptive organ implies that investigating a particular brain region in isolation is insufficient to understand how the brain works. While knowing structure and volume of individual brain regions in both health and diseases is critical, it is no longer enough. Rather, knowing how individual structures are connected anatomically and functionally to other brain regions and networks is required, as is knowing the myriad of different configurations brain networks can take in response to incoming internal and external information and in adapting to predicted needs (11). Further, the adaptive brain's integration of interoception, exteroception, emotion, networks such as the DMN suggests that homeostatic and allostatic mechanisms and emotion require integration and inclusion into current models of understanding brain function in both health and disease.

Findings showing that the brain is highly adaptive provide for new theoretical and research models that consider interdependent brain networks, prediction, minimization of prediction errors, and active inference. Evidence implicating the insular cortex, the cingulate cortex, and other frontal regions as elements of interdependent brain regions in integrating interoceptive and exteroceptive input and providing predictions of future homeostatic and allostatic needs illuminates these brain regions and their connections as important regions of interest in functional imaging studies, albeit in relationship to other regions and brain circuits. And, finally, the integration of internal and external information from exteroceptive and interoceptive nerves and regions with predictions and adaptations in the brain and adaptive integration between incoming and outgoing information in some ways might make distinctions between the peripheral nervous system and the central nervous system obsolete (11).

Because a key aspect of the brain is interdependence across multiple networks to optimize adaption to changing internal and external environments, it is important to consider factors that can decrease the brain's adaptability and how a decrease in adaptivity might affect brain function. Anything that impairs the brain's ability to adapt can become critical in understanding putative reasons for impaired adaptability, including conditions such as mental illness (59). For example, among the other adverse effects of chronic stress exposure is the reduced ability to adapt to stress, resulting in a cycle in which stress impedes an animal's ability to appropriately respond to stress (60).

An important implication, therefore, of focusing on the brain's ability to adapt to stress and to adapt to and to predict continuously changing external and internal states is that when the brain's adaptive and predictive systems are not functioning properly, disease states could result (61). Genetic, epigenetic, environmental, and stochastic insults to the frontal, cingulate, and insular interoceptive systems and their output connections that predict and adjust autonomic, hormonal, and immunologic needs and responses, respectively, have the potential to result in disease. Barrett and Simmons (29) argue that improper function of these brain regions interferes with the regulation of the hypothalamic-pituitary-adrenal axis and can lead to depression and to a proinflammatory state. The frontal interoceptive system and the insula enable brain adaptation to changing environmental and internal circumstances and needs to maintain homeostasis and to initiate allostasis. In essence, the brain interoceptive center promotes adaption to ever changing internal and external circumstances through prediction and subsequent adaptation. Impaired adaptive function in these brain regions, therefore, could result in disease, and inadequate active inference could result in mental illness such as panic and depressive disorders (35).

Behavioral dispositional negativity is a condition that appears to be a vulnerability factor for a variety of psychopathological conditions (20). Both genetic and environmental factors appear to be associated with the development of dispositional negativity, and dispositional negativity appears associated with abnormal function in several brain regions, including the insula, amygdala, mid-cingulate, and orbitofrontal cortex, brain regions that overlap with networks involved with prediction and adaptation. Possibly developing as initially adaptive processing after multiple threats, dispositional negativity appears to be associated with truncating input to the brain and interference with error-prediction reduction, ultimately resulting in worse error prediction. While the truncation of error processing might be adaptive initially in that it makes the environment seem more predictable, it becomes maladaptive in the long term as worsening prediction errors impede brain adaptability (20). Accordingly, factors that affect brain regions involved in prediction error have the potential to result in some types of psychopathologies such as depression, anxiety (20, 29), fatigue, and autism-spectrum disorders (30).

In addition to associations between dispositional negativity and psychopathology, abnormal functioning in brain regions such as the insula that are involved in prediction and rapid adaptation to continually changing internal states is implicated in the pathogenesis of several anxiety disorders, as well as with obsessive-compulsive disorder. Interceptive information via the glossopharyngeal and vagal nerves reaches the insula and other regions and networks that are involved in interoception. Abnormal function in these regions including the insula appear have been associated with some anxiety disorders and with obsessive compulsive disorder (62). Given the associations between abnormal function in the insula and other regions with some anxiety disorders and with obsessive-compulsive disorder (62) and findings showing the involvement of the insula with error prediction and minimization in influencing rapid adaptation to changing environments (29), it is plausible that abnormal function in adaptation vis-a-vi error prediction is directly associated with some anxiety and obsessive-compulsive disorders, although much additional work is required to identify the brain networks involved with insula function Abnormalities in brain energy function have been associated with autism-spectrum disorders (63), which could be involved with the importance of maintaining and predicting energy needs in the brain in its overall adaptivity (17), implicating dysfunction in the brain adaptivity as possibly associated with some schizophrenia-spectrum and autism-spectrum disorders. Further, functional neuroimaging of individuals with schizophrenia-spectrum, autism-spectrum disorders, and anxiety disorders implicates structural and functional abnormalities of salience networks, disordering how the individual can adapt to changing interoceptive and exteroceptive input, and interfering with the brain's ability to learn functional error prediction algorithms (64). Together, these findings suggest the possibility that dysfunction in brain regions involved in interoception, error prediction, and adaptation affect the pathogenesis of several different types of psychopathologies.

Similar implications arise when considering how malfunction in brain regions involved in error processing possibly might affect function of organ systems in addition to the brain, intimately linking impaired adaptive processing in brain regions involved in interoception, exteroception, prediction, and adaptation to disease in other organ systems. Because brain regions involved in interoception are predicting physiological parameters such as blood glucose concentration, immune states, and heart rate, abnormalities in brain regions involved in interoception and error prediction could be associated with other diseases, such as obesity and diabetes (29). Associations between improperly functioning interoceptive regions such as the insula and related networks provide a common neurological basis to not only some psychiatric disorders but also to some other diseases (29), possibly leading to a novel and more basic understanding of disease and linking psychiatric and medical diseases to dysfunction in the same brain regions and to overall decreased brain adaptiveness.

Both interoceptive and emotional processing with their associated allostatic adaptation appear to rely on predictive coding, wherein the brain including the insula considers previous internal and external conditions in making adaptive changes. Showing how abnormalities in predictive interoceptive and emotional function might be related to other diseases, emotional ability in understanding emotions, interoceptive awareness, and associated activation in the anterior insula were associated with brain white-matter microstructure, providing evidence that abnormalities in adaptation based on faulty predictive coding could result in white-matter disease (65).

### Clinical Implications

Given the associations between prediction errors, adaptation, and disease, adaptive brain theory may provide a framework for the understanding and treatment of mental illness (20, 33). Despite significant progress in neuroscience research including information about brain and genetic abnormalities associated with psychiatric disorders and over the last several decades, specifically efforts to advance the prevention and cure of mental illness, little headway has been made (66). Approaching the brain from a triune-brain perspective or similar viewpoints may contribute to hypotheses that are built on inaccurate assumptions about brain functioning. Kozak and Cuthbert (67) note that “there is thus an a priori assumption that the diagnoses refer to real disorders, with ensuing assumptions that they involve a unitary pathophysiology and psychopathology and that the task of a science of disorders is to find the underlying biology of the specific disease entities… [but these] assumptions now [appear] to be false… these approaches have failed to produce significant advances in the understanding or treatment of mental disorders” (p. 287).

To address the disconnection between mental-health diagnoses and neuroscience findings, the NIH initiated a research program, the Research Domain Criteria (RDoC), that uses a dimensional system based upon observable behavior and neurobiological measures, integrating psychology, biology, and neuroscientific findings (66). The key systems under study are negative valence systems, positive valence systems, cognitive systems, systems for social processes, and arousal/modulatory systems. These systems fit well with LeDoux's (8) approach of ‘survival circuits' underlying emotional responses, Barrett's (11) constructionist approach to the creation of emotions addressing underlying physiological needs, and Lang's (68) psychophysiological approach to affect and motivation. By combining current neuroscience findings within a broader dimensional framework of mental health, there is potential to improve prevention and treatment of mental illness. Adaptive brain theory builds upon these concepts, providing a theoretically sound framework for understanding mental health and generating effective hypotheses. Research on the neural impact of psychotherapy nicely illustrates how the adaptative brain is malleable and is impacted by treatment for excessive fear and threat responses. For example, MRI studies of patients suffering from panic disorder undergoing CBT show altered brain functioning and decreased fear responses (69, 70). Similarly, CBT for psychosis shows decreased activation in neural circuits involved in threat responses after successful treatment (71).

## Conclusion

A primary function of the brain is to make adaptive models (20, 35) of the external and internal environments. Current findings indicate that brain function is based on interdependent networks in contrast to earlier conceptions such as the triune brain in which hypothesized distinct brain centers operated relatively independently of each other. In particular, the brain appears to work by integrating interoceptive and exteroceptive information to make predictions about future metabolic, energy, and other needs while it adapts to continually changing external and internal conditions to maintain homeostasis and to initiate allostasis as needed. As part of this adaptive process, the brain then compares predictions with incoming information and makes adjustment to minimize error prediction further promoting adaptation and health. The brain also might make predictions about potential outcomes from a variety of different possible actions using active inference (33). A triune-brain framework limits understanding of pathophysiology. Conceptualization of the brain's role in adaptation provides new theoretical and clinical insights into brain function in both health and disease. Improper function of brain regions such as the insula and prefrontal cortex and their associated networks leading to impaired adaption and dysregulated affect might be associated with conditions such as depression, anxiety, schizophrenia, and other disease states, possibly indicating an expanded role of the brain in the pathophysiology of disease and providing novel insights into the nature of some diseases as well as potentially identifying and developing new treatment approaches.

## Author Contributions

All authors listed have made a substantial, direct, and intellectual contribution to the work and approved it for publication.

## Conflict of Interest

The authors declare that the research was conducted in the absence of any commercial or financial relationships that could be construed as a potential conflict of interest.

## Publisher's Note

All claims expressed in this article are solely those of the authors and do not necessarily represent those of their affiliated organizations, or those of the publisher, the editors and the reviewers. Any product that may be evaluated in this article, or claim that may be made by its manufacturer, is not guaranteed or endorsed by the publisher.

## References

[1] Cory GA. From Maclean's Triune Brain concept to the conflict systems neurobehavioral model: the subjective basis of moral and spiritual consciousness. Zygon J Relig Sci. (2000) 35:385. 10.1111/0591-2385.00283

[2] MacLean PD. The Triune Brain in Evolution: Role in Paleocerebral Functions. New York, NY: Springer (1990). 10.1126/science.250.4978.303-a 17797318

[3] MacLean PD. Paul D. Maclean. In: Squire LR, editor. The History of Neuroscience in Autobiography. London: Academic Press (1998) p. 244–75. 10.1016/S1874-6055(99)80011-5

[4] Panksepp J Moskal JR Panksepp JB Kroes RA. Comparative approaches in evolutionary psychology: molecular neuroscience meets the mind. Neuro Endocrinol Lett. (2002) 23(Suppl. 4):105–15. 12496741

[5] Heimer L Van Hoesen GW. The limbic lobe and its output channels: implications for emotional functions and adaptive behavior. Neurosci Biobehav Rev. (2006) 30:126–47. 10.1016/j.neubiorev.2005.06.006 16183121

[6] Cesario J Johnson D Eisthen H. Your brain is not an onion with a tiny reptile inside. Curr Direct Psychol Sci. (2020) 29:255–60. 10.1177/0963721420917687

[7] Striedter GF. Principles of Brain Evolution. Sunderland, MA: Sinauer Associates (2005). 10.1016/B978-012547626-3/50002-8

[8] LeDoux J. Rethinking the emotional brain. Neuron. (2012) 73:653–76. 10.1016/j.neuron.2012.02.004 22365542 PMC3625946

[9] Bush G Vogt BA Holmes J Dale AM Greve D Jenike MA. Dorsal anterior cingulate cortex: a role in reward-based decision making. Proc Natl Acad Sci USA. (2002) 99:523–8. 10.1073/pnas.012470999 11756669 PMC117593

[10] Shackman AJ Salomons TV Slagter HA Fox AS Winter JJ Davidson RJ. The integration of negative affect, pain and cognitive control in the cingulate cortex. Nat Rev Neurosci. (2011) 12:154–67. 10.1038/nrn2994 21331082 PMC3044650

[11] Barrett LF. The theory of constructed emotion: an active inference account of interoception and categorization. Soc Cogn Affect Neurosci. (2017) 12:1–23. 10.1093/scan/nsx060 27798257 PMC5390700

[12] Raichle ME. The brain's default mode network. Annu Rev Neurosci. (2015) 38:433–47. 10.1146/annurev-neuro-071013-014030 25938726

[13] Anticevic A Repovs G Corlett PR Barch DM. Negative and nonemotional interference with visual working memory in schizophrenia. Biol Psychiatry. (2011) 70:1159–68. 10.1016/j.biopsych.2011.07.010 21861986

[14] Corbetta M Patel G Shulman GL. The reorienting system of the human brain: from environment to theory of mind. Neuron. (2008) 58:306–24. 10.1016/j.neuron.2008.04.017 18466742 PMC2441869

[15] Fox MD Snyder AZ Vincent JL Corbetta M Van Essen DC Raichle ME. The human brain is intrinsically organized into dynamic, anticorrelated functional networks. Proc Natl Acad Sci U S A. (2005) 102:9673–8. 10.1073/pnas.0504136102 15976020 PMC1157105

[16] Raichle ME MacLeod AM Snyder AZ Powers WJ Gusnard DA Shulman GL. A default mode of brain function. Proc Natl Acad Sci U S A. (2001) 98:676–82. 10.1073/pnas.98.2.676 11209064 PMC14647

[17] Quigley KS Kanoski S Grill WM Barrett LF Tsakiris M. Functions of interoception: from energy regulation to experience of self. Trends Neurosci. (2021) 44:29–38. 10.1016/j.tins.2020.09.008 33378654 PMC7780233

[18] Brossschot JF Verkuil B Thayer JF. Generalized Unsafety Theory of Stress: unsafe environments and conditions, and the default stress response. Int J Environ Res Public Health. (2018) 15:464. 10.3390/ijerph15030464 29518937 PMC5877009

[19] Thayer JF Lane RD. Claude Bernard and the heart-brain connection: further elaboration of a model of neurovisceral integration. Neurosci Biobehav Rev. (2009) 33:81–8. 10.1016/j.neubiorev.2008.08.004 18771686

[20] Van den Bergh O Brosschot J Critchley H Thayer JF Ottaviani C. Better safe than sorry: a common signature of general vulnerability for psychopathology. Perspectives on Psychol Sci. (2021) 16:225–46. 10.1177/1745691620950690 33006909

[21] Bowlby J. Attachment and Loss. New York, NY: Basic Books (1980).

[22] Schulkin J. Social allostasis: anticipatory regulation of the internal milieu. Front Evolut Neurosc. (2011) 2:111. 10.3389/fnevo.2010.00111 21369352 PMC3037529

[23] Schulkin J Sterling P. Allostasis: a brain-centered, predictive mode of physiological regulation. Trends Neurosci. (2019) 42:740–52. 10.1016/j.tins.2019.07.010 31488322

[24] Raglan GB Schulkin J. Introduction to allostasis and allostatic load. In: Kent M, Davis MC, Reich JW, editors. The Resilience Handbook: Approaches to Stress and Trauma. Routledge: Taylor and Francis Group (2014). p. 44–52.

[25] Sterling P. Principles of allostasis: optimal design, predictive regulation, pathophysiology, and rational therapeutics. In: Schulkin J, editor. Allostasis, Homeostasis, and the Costs of Physiological Adaptation. Cambridge: Cambridge University Press (2004). p. 17–64. 10.1017/CBO9781316257081.004

[26] Bar M. Predictions: a universal principle in the operation of the human brain. Philos Trans R Soc. (2009) 364:1181–2. 10.1098/rstb.2008.0321 19527998 PMC2666718

[27] Clark A. Whatever next? Predictive brains, situated agents, and the future of cognitive science. Behav Brain Sci. (2013) 36:181–253. 10.1017/S0140525X12000477 23663408

[28] Clark A. Radical predictive processing. South J Philos. (2015) 53:3–27. 10.1111/sjp.12120

[29] Barrett LF Simmons WK. Interoceptive predictions in the brain. Nat Rev Neurosci. (2015) 16:419–29. 10.1038/nrn3950 26016744 PMC4731102

[30] Seth AK Friston KJ. Active interoceptive inference and the emotional brain. Philos Trans R Soc B. (2016) 371:20160007. 10.1098/rstb.2016.0007 28080966 PMC5062097

[31] Menon V Uddin LQ. Saliency, switching, attention and control: a network model of insula function. Brain Struct Funct. (2010) 214:655–67. 10.1007/s00429-010-0262-0 20512370 PMC2899886

[32] Hesp C Smith R Parr T Allen M Friston KJ Ramstead MJD. Deeply felt affect: the emergence of valence in deep active inference. Neural Comput. (2021) 33:398–446. 10.1162/neco_a_01341 33253028 PMC8594962

[33] Smith R Badcock P Friston KJ. Recent advances in the application of predictive coding and active inference models within clinical neuroscience. Psychiatry Clin Neurosci. (2021) 75:3–13. 10.1111/pcn.13138 32860285

[34] Pezzulo G Rigoli F Friston KJ. Active inference, homeostatic regulation and adaptive behavioural control. Progress Neurobiol. (2015) 134:17–35. 10.1016/j.pneurobio.2015.09.001 26365173 PMC4779150

[35] Paulus MP Feinstein JS Khalsa SS. An active inference approach to interoceptive psychopathology. Annu Rev Clin Psychol. (2019) 15:97–122. 10.1146/annurev-clinpsy-050718-095617 31067416 PMC7280559

[36] Yeshurun Y Nguyen M Hasson U. The default mode network: where the idiosyncratic self meets the shared social world. Nature Rev Neurosci. (2021) 22:181–92. 10.1038/s41583-020-00420-w 33483717 PMC7959111

[37] Beucke JC Sepulcre J Eldaief MC Sebold M Kathmann N Kaufmann C. Default mode network subsystem alterations in obsessive-compulsive disorder. Br J Psychiatry. (2014) 205:376–82. 10.1192/bjp.bp.113.137380 25257066

[38] Tozzi L Zhang X Chesnut M Holt-Gosselin B Ramirez CA Williams LM. Reduced functional connectivity of default mode network subsystems in depression: meta-analytic evidence and relationship with trait rumination. Neuroimage Clin. (2021) 30:102570. 10.1016/j.nicl.2021.102570 33540370 PMC7856327

[39] Sridharan D Levitin DJ Menon V. A critical role for the right fronto-insular cortex in switching between central-executive and default-mode networks. Proc Natl Acad Sci. (2008) 105:12569–74. 10.1073/pnas.0800005105 18723676 PMC2527952

[40] Vatansever D Menon DK Stamatakis EA. Default mode contributions to automated information processing. Proc Natl Acad Sci. (2017). 114: 12821–6. 10.1073/pnas.1710521114 PMC5715758 29078345

[41] Craig AD. How do you feel – now? The anterior insula and human awareness. Nat Rev Neurosci. (2009) 10:59–70. 10.1038/nrn2555 19096369

[42] Rolls ET. Cerebral Cortex: Principles of Operation. Oxford: Oxford University Press (2016). 10.1093/acprof:oso/9780198784852.001.0001

[43] Barrett LF Bliss-Moreau E. Affect as a psychological primitive. Adv Exp Soc Psychol. (2009) 41:167–218. 10.1016/S0065-2601(08)00404-8 20552040 PMC2884406

[44] Lang PJ Bradley MM. Emotion and the motivational brain. Biol Psychol. (2010) 84:437–50. 10.1016/j.biopsycho.2009.10.007 19879918 PMC3612949

[45] Tooby J Cosmides L. The psychological foundations of culture. In: Barkow JH, Cosmides L, Tooby J, editors.The Adapted Mind: Evolutionary Psychology and the Generation of Culture. Oxford: Oxford University Press (1992). p. 19–136.

[46] Barrett LF Bar M. See it with feeling: affective predictions during object perception. Philos Trans R Soc. (2009) 364:1325–34. 10.1098/rstb.2008.0312 19528014 PMC2666711

[47] Storbeck J Clore GL. On the interdependence of cognition and emotion. Cogn Emot. (2007) 21:1212–37. 10.1080/02699930701438020 18458789 PMC2366118

[48] Lang PJ Bradley MM Cuthbert BN. Emotion, attention, and the startle reflex. Psychol Rev. (1990) 97:377–95. 10.1037/0033-295X.97.3.377 2200076

[49] Russell JA. Core affect and the psychological construction of emotion. Psychol Rev. (2003) 110:145–72. 10.1037/0033-295X.110.1.145 12529060

[50] Clore GL Huntsinger JR. How the object of affect guides its impact. Emot Rev. (2009) 1:39–54. 10.1177/1754073908097185 25431618 PMC4243521

[51] Van de Cruys S. Affective value in the predictive mind. In: Metzinger T Wiese W, editors. Philosophy and Predictive Processing: 24. Frankfurt am Main: MIND Group (2017).

[52] Clore GL Schiller AJ Shaked A. Affect and cognition: three principles. Curr Opin Behav Sci. (2018) 19:78–82. 10.1016/j.cobeha.2017.11.010 30271831 PMC6159898

[53] LeDoux JE Damasio AR. Emotions and feelings. In: Kandel ER, Schwartz JH, Jessell TM, Siegelbaum SA, Hudspeth AJ, editors. Principles of Neural Science. 5th ed. New York, NY: McGraw Hill (2013).

[54] Hagen EH Symons D. Natural psychology: the environment of evolutionary adaptedness and the structure of cognition.In: Gangestad SW, Simpson JA, editors. The Evolution of Mind: Fundamental Questions and Controversies. The Guilford Press (2007). p. 38–44.

[55] Buss DM. Human nature and individual differences: evolution of human personality. In: John OP, Robins RW, Pervin LA, editors. Handbook of Personality: Theory and Research. The Guilford Press (2008). p 29–60.

[56] Hogan R Sherman RA. Personality theory and the nature of human nature. Pers Individ Dif. (2020) 152:1–5. 10.1016/j.paid.2019.109561

[57] Hogan R Bond MH. Culture and personality. In: Corr PJ, Matthews G, editors. The Cambridge Handbook for Personality Psychology. New York, NY: Cambridge University Press (2009). p. 577–88. 10.1017/CBO9780511596544.036

[58] Baumeister RF Leary MR. The need to belong: desire for interpersonal attachments as a fundamental human motivation. Psychol Bull. (1995) 117:497–529. 10.1037/0033-2909.117.3.497 7777651

[59] Van de Cruys S Van Dessel P. Mental distress through the prism of predictive processing theory. Curr Opin Psychol. (2021) 41:107–12. 10.1016/j.copsyc.2021.07.006 34388670

[60] Zhang W Hashemi MM Kaldewaij R Koch S Beckmann C Klumpers F. Acute stress alters the 'default' brain processing. Neuroimage. (2019) 189:870–7. 10.1016/j.neuroimage.2019.01.063 30703518

[61] Lynn SK Barrett LF. “Utilizing” signal detection theory. Psychol Sci. (2014) 25:1663–73. 10.1177/0956797614541991 25097061 PMC4304641

[62] Stern ER. Neural circuitry of interoception: new insights into anxiety and obsessive- compulsive disorders. Curr Treat Options Psychiatry. (2014) 1:235–47. 10.1007/s40501-014-0019-0 33344105 PMC7747958

[63] Gordon A Forsingdal A Klewe IV Nielsen J Didriksen M Werge T. Transcriptomic networks implicate neuronal energetic abnormalities in three mouse models harboring autism and schizophrenia-associated mutations. Mol Psychiatry. (2021) 26:1520–34. 10.1038/s41380-019-0576-0 31705054

[64] Menon V. Large-scale brain networks and psychopathology: a unifying triple network model. Trends Cogn Sci. (2011) 15:483–506. 10.1016/j.tics.2011.08.003 21908230

[65] Dobrushina OR Arina GA Dobrynina LA Suslina AD Solodchik PO Belopasova AV. The ability to understand emotions is associated with interoception-related insular activation and white-matter integrity during aging. Psychophysiology. (2020) 57:e13537. 10.1111/psyp.13537 31994733

[66] Cuthbert BN Insel TR. Toward the future of psychiatric diagnosis: the seven pillars of RDoC. BMC Med. (2013) 11:126. 10.1186/1741-7015-11-126 23672542 PMC3653747

[67] Kozak MJ Cuthbert BN. The NIMH research domain criteria initiative: background, issues, and pragmatics. Psychophysiology. (2016) 53:286–97. 10.1111/psyp.12518 26877115

[68] Lang PJ McTeague LM Bradley MM. The psychophysiology of anxiety and mood disorders: the RDoC challenge. Zeitschrift Psychol. (2017) 225:175–88. 10.1027/2151-2604/a000302

[69] Straube B Lueken U Jansen A Konrad C Gloster ATT Gerlach ALL. Neural correlates of procedural variants in cognitive-behavioral therapy: a randomized, controlled multicenter fMRI study. Psychother Psychosom. (2014) 83:222–33. 10.1159/000359955 24970601

[70] Yang Y Lueken U Richter J Hamm A Wittmann A Konrad C. Effect of CBT on biased semantic network in panic disorder: a multicenter fMRI study using semantic priming. Am J Psychiatry. (2020) 177:254–64. 10.1176/appi.ajp.2019.19020202 31838872

[71] Kumari V Fannon D Peters ER Ffytche DH Sumich AL Premkumar P. Neural changes following cognitive behaviour therapy for psychosis: a longitudinal study. Brain. (2011) 134:2396–407. 10.1093/brain/awr154 21772062 PMC3155705

